# Relationship between elimination disorders and internalizing-externalizing problems in children: A systematic review and meta-analysis

**DOI:** 10.1192/j.eurpsy.2023.723

**Published:** 2023-07-19

**Authors:** C. Aymerich, B. Pedruzo, M. Pacho, M. Laborda, J. Herrero, M. Bordenave, G. Salazar de Pablo, E. Sesma, A. Fernandez-Rivas, A. Catalan, M. Á. González-Torres

**Affiliations:** ^1^Psychiatry, Basurto University Hospital, Bilbao, Spain; ^2^ Institute of Psychiatry, Psychology and Neuroscience, King’s College London; ^3^Child and Adolescent Mental Health Services, South London and Maudsley NHS Foundation Trust, London, United Kingdom; ^4^Neuroscience, University of the Basque Country (UPV/EHU); ^5^Psychiatry, Biocruces Bizkaia Health Research Institute, OSI Bilbao-Basurto, Bilbao; ^6^Centro de Investigación en Red de Salud Mental (CIBERSAM), Madrid; ^7^Neuroscience, University of the Basque Country (UPV/EHU), Leioa, Spain; ^8^Psychosis Studies, Institute of Psychiatry, Psychology and Neuroscience, King’s College London, London, United Kingdom

## Abstract

**Introduction:**

Elimination disorders (ED) include enuresis, defined as wetting from 5 years, and encopresis, defined as soiling from 4 years onwards after organic causes are excluded. They are highly prevalent in childhood and often associated with clinically relevant comorbid psychological disorders. However, no systematic review or meta-analysis examines their co-occurrence with internalizing and externalizing problems in children.

**Objectives:**

The aim of this study is to determine if, and to what extent, children with ED show higher internalizing and externalizing problems than their healthy peers.

**Methods:**

A multistep literature search was performed from database inception until May 1st, 2022. PRISMA/MOOSE-compliant systematic review (PROSPERO: CRD42022303555) were used to identify studies reporting on internalizing and/or externalizing symptoms in children with an ED and a healthy control (HC) group. First, a systematic review was provided. Second, where data allowed for it, a quantitative meta-analysis using random effects model was conducted to analyze the differences between the ED and the HC groups for internalizing and externalizing symptoms. Effect size was standardized mean difference. Meta-regression analyses were conducted to examine the effect of sex, age, and study quality. Funnel plots were used to detect a publication bias. Where found, the trim and fill method was used to correct it.

**Results:**

36 articles were included, 32 of them reporting on enuresis (n=3244; mean age=9.4; SD=3.4; 43.84% female) and 7 of them on encopresis (n=214; mean age=8.6; SD=2.3; 36.24% female) [Image 1]. The ED group presented significantly lower self-concept (ES:0.42; 95%CI: [0.08;9.76]; p=0.017) and higher symptom scores for thought problems (ES:-0.26; 95%CI: [-0.43;-0.09]; p=0.003), externalizing symptoms (ES:-0.20; 95%CI: [-0.37;-0.03]; p=0.020), attention problems (ES:-0.37; 95%CI: [-0.51;-0.22]; p=0.0001), aggressive behaviour (ES:-0.33; 95%CI: [-0.62;-0.04]; p=0.025) and social problems (ES: 0.39; 95%CI: [-0.58;-0.21]; p=0.0001) [Image 2]. Significant publication biases were found across several of the studied domains [Image 3]. No significant effect of sex, age or quality of the study score was found.

**Image:**

**Figure:**
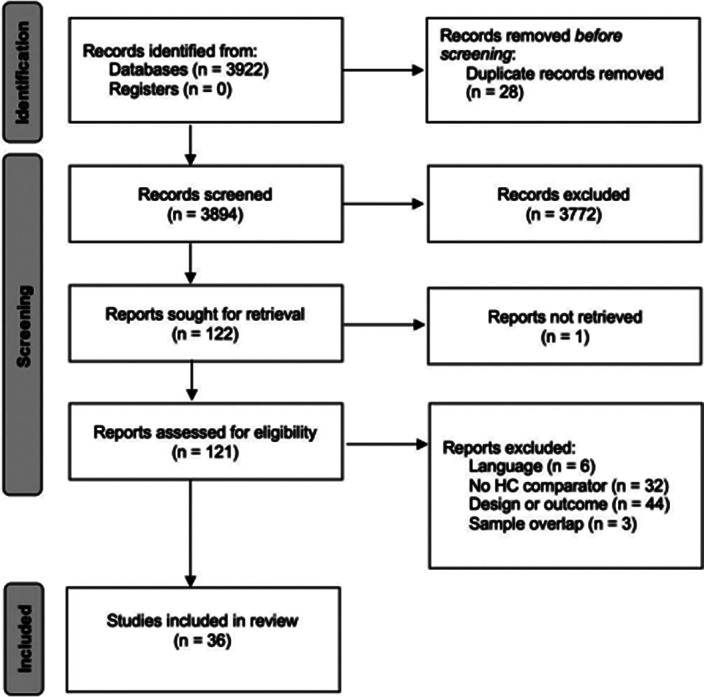

**Image 2:**

**Figure:**
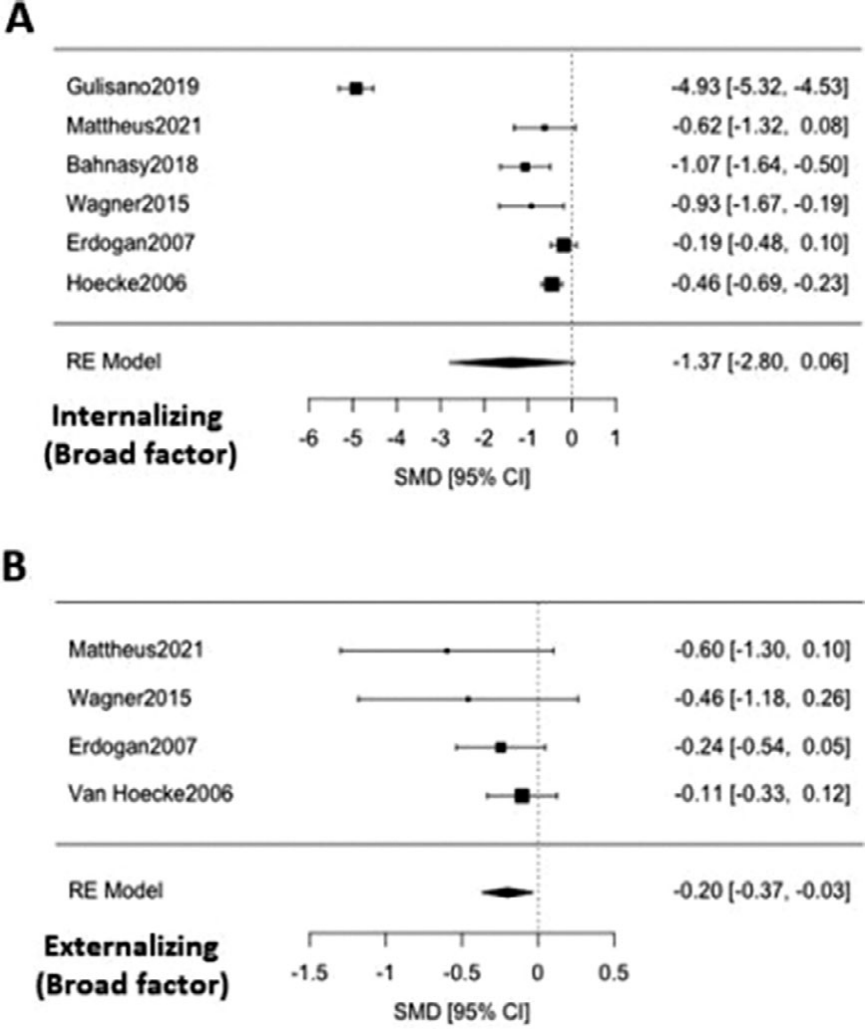

**Image 3:**

**Figure:**
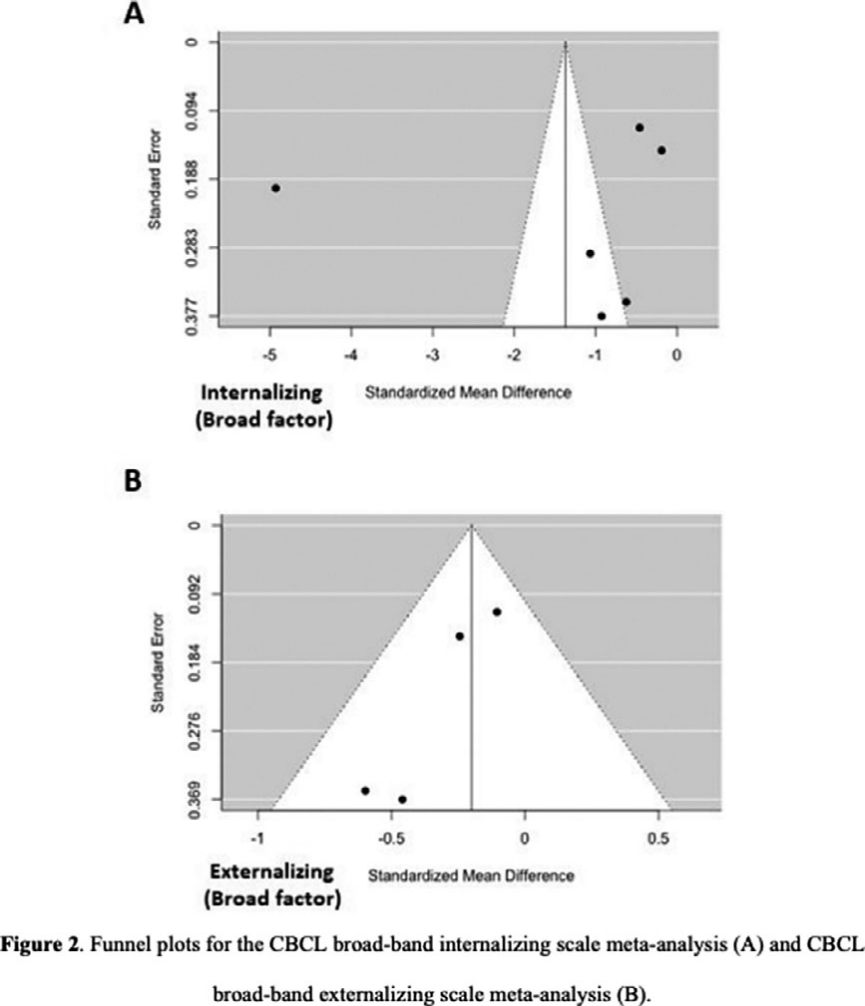

**Conclusions:**

Children with an elimination disorder may have significant internalizing and externalizing problems, as well as impaired self-concept. It is recommendable to screen for them in children with ED and provide interventions as appropriate.

**Disclosure of Interest:**

None Declared

